# Rehearsing post‐Covid‐19 citizenship: Social representations of UK Covid‐19 mutual aid

**DOI:** 10.1111/bjso.12535

**Published:** 2022-03-29

**Authors:** Emma O’Dwyer, Luiz Gustavo Silva Souza, Neus Beascoechea‐Seguí

**Affiliations:** ^1^ Kingston University London London UK; ^2^ Fluminense Federal University Niterói Brazil; ^3^ Independent Researcher London UK

**Keywords:** citizenship, community response, Covid‐19, mutual aid

## Abstract

People across the world have responded to the pandemic by mobilizing and organizing to support their communities, setting up mutual aid groups to provide practical, financial, and social support. Mutual aid means short‐term 'crisis response' for some, while for other groups, it is a chance to radically restructure society, and what it means to be a member of that society. Drawing on social representations theory and previous work on citizenship in social and political psychology, we examined the ways in which mutual aid was understood and performed by members of UK Covid‐19 mutual aid groups. We conducted 29 interviews with members of these groups in May/June 2020. A reflexive thematic analysis showed that mutual aid groups were characterized as complex, efficient, and non‐hierarchical units, operating on the principles of solidarity, kindness, and trust. Two tensions were evident in the data, specifically between (1) collaboration with existing organizations and structures (e.g., local government and the police), and resistance to it and (2) maximizing group inclusivity and sustaining political critique. Findings are discussed in relation to existing theoretical and empirical work on citizenship and mutual aid groups.

## BACKGROUND

Previous work in social and political psychology has examined the ways in which people construct, enact, and claim citizenship in a diverse range of contexts. This work emphasizes the need to develop understanding of the term from the bottom‐up, as it is mobilized and oriented to in people's everyday lives (Condor, 2011), often performed outside the sphere of formal politics or state‐sanctioned actions (Isin, 2009; Miraftab, 2004). This work naturally aligns itself with a social representations approach which specifies a dynamic relationship between social practices and everyday sense‐making (Moscovici, 1961). The Covid‐19 pandemic, as an “emergent form” (Andreouli et al., 2019), is fundamentally disruptive to common sense and everyday lives and as such, leaves open the possibility of social and political change. In the present study, we attend to the potential impact of the crisis on lay understandings and enactments of citizenship by focusing on the phenomenon of Covid‐19 mutual aid groups in the United Kingdom. We conceptualize these groups as spaces in which new understandings and forms of citizenship may be developed, rehearsed, and enacted, and thus which may have implications for future civic and political engagement in the United Kingdom.

### Understandings of citizenship

Citizenship has been theorized in diverse ways in the interdisciplinary field of citizenship studies. Three key conceptualizations dominate, which understand citizenship as liberal, communitarian, or civic republican (Langhout & Fernández, 2016; Lister, 1998). Liberal perspectives (Marshall, 1950) define citizenship as a status stemming from an *individual's* rights and responsibilities towards the state. The ideal of universal citizenship embedded in this model has been heavily critiqued. For example, Young (1989, p. 251) advocated for a “differentiated citizenship” which recognized the difference and did not mask the unequal status of different groups. In contrast to the individualism of liberal perspectives, communitarian approaches (Etzioni, 1993) define citizenship as tied to communities defined by shared values and group identities, rather than communities functioning as an aggregate of individuals as per the liberal model (Delanty, 2002). Civic republican models (Oldfield, 1994) conceptualize citizenship as a practice—a duty to actively participate in civil society and work towards a public good—which also works to sustain and construct communities. However, scholars have also challenged this perspective due to its potential exclusion of women, sexual, and ethnic minorities (Lister, 1997; Young, 1989).

Current conceptualizations of citizenship proliferate; work has focused on cultural citizenship (Langhout & Fernández, 2016; Rosaldo, 1994) multicultural citizenship (Kymlicka, 1995), biological citizenship (Rose & Novas, 2005), to name a few. A relevant example here is Isin and colleagues’ work on “acts of citizenship” (Isin, 2017; Isin & Nielsen, 2008). This approach moves away from state‐centric accounts of citizenship and instead examines performances of citizenship in relation to social relations and subjectivities. Citizenship is bound up with social struggle, first for rights to be recognized as such, and second over the breadth and depth of these rights (Isin & Nyers, 2014). Thus, the practice of citizenship is not only about obeying or adhering to a body of rules but also opposition (Isin, 2017; Kostakopoulou, 2008) and resistance (Passini & Morselli, 2011). Relatedly, Miraftab (2004) distinguishes between the capacity of those working within informal politics to flexibly occupy the “invited” or “invented” spaces of citizenship, where the former denotes state‐sanctioned grassroots action (i.e., through funding and collaboration with local authorities) to *survive* hardships and inequalities, and the latter refers to grassroots action to *resist* continued hardships and inequalities which are independent of formal structures.

Work has also interrogated the relationship between neoliberal capitalism and citizenship (Changfoot, 2007; Ong, 2006; Sparke, 2006). Neoliberalism is defined here as “the new political, economic, and social arrangements within society that emphasise market relations, re‐tasking the role of the state, and individual responsibility” (Springer et al., 2016, p. 2). In this context, citizens are recast as consumers (Clarke et al., 2007). Illustrative of this, Sparke’s (2006) analysis of an expedited border‐crossing program in Canada illuminated the contrast between the paradigmatic neoliberal consumerist citizen whose right to cross borders is buttressed by free trade and related laws, and borderless citizens whose realities are characterized by extraordinary rendition and expedited removal, who occupy “a world without constitution” (p. 174). Also foregrounding mobility, Mitchell (2016) noted that neoliberalism prefers a citizen who “lacks deep allegiance to a single locale, operating rather as an entity responsible to the networks and flows of the system itself” (p. 108), which has negative consequences for practices related to culture, health, and responsibility towards others and the environment.

### Social psychological accounts of citizenship

Instigating social psychological inquiry into citizenship, Shotter (1993) conceptualized citizenship as practice, and highlighted the way in which individuals rhetorically accomplish a sense of identity and belonging in the politics of everyday social life. Subsequently, other researchers have also approached citizenship as a dynamic, negotiated, and situated practice, adopting discursive (Barnes et al., 2004; Kadianaki et al., 2020), rhetorical (Andreouli & Brice, 2021; Andreouli & Dashtipour, 2014; Condor & Gibson, 2007), and social representations approaches (Kadianaki & Andreouli, 2017; Rodríguez López et al., 2015). While there are significant differences between each of these approaches in terms of epistemology and methodology (see Batel & Castro, 2018 for a recent relevant discussion), all attempt to move past a state‐centric account of citizenship and foreground processes of meaning, negotiation, enactment, and contestation, in relation to citizenship.

Empirical work has investigated how people construct, enact, and claim citizenship in everyday contexts, for example amongst UK citizenship officers (Andreouli & Dashtipour, 2014) and indigenous and non‐indigenous Greeks after significant changes to national government legislation (Andreouli et al., 2016). For example, using a social representations approach, Rodríguez López et al. (2015) examined the relationship between re‐integration and citizenship among ex‐combatants in Colombia and found that the practice of citizenship was influenced and constrained by civil society in Colombia as well as the state, particularly through its acknowledgement via official documentation.

Relatedly, work has underscored the way in which constructions of citizenship are bound up with power dynamics, particularly serving to position some people as legitimate subjects while others are excluded. This is evident in studies that have examined constructions of citizenship in government discourse, for example, the UK citizenship test (Gray & Griffin, 2014), UK immigration policy documents (Andreouli & Howarth, 2013), and how sexual citizenship was constructed in Greek Cypriot media in response to a civil union law (Kadianaki et al., 2020). Particularly relevant here, Andreouli and Brice’s (2021) analysis of UK government rhetoric during the early stages of the Covid‐19 pandemic (March–November 2020) identified five models of citizenship—the confined, heroic, sacrificial, unfree, and responsible citizen. Individuals were repeatedly constructed as personally responsible for public health during “lockdown,” and responsible for economic recovery in its aftermath. In a break from the neoliberal status quo, government actors emphasized the importance of other‐focused values and actions but reverted to defining citizenship in terms of economic output and consumption as restrictions were loosened.

### Social representations theory

Following previous work (Kadianaki & Andreouli, 2017; Rodríguez López et al., 2015) we adopt a social representations approach to investigate the phenomenon of Covid‐19 mutual aid. A social representation can be defined as the way in which a group socially constructs the “reality” of a certain object, referring at the same time to the process of construction and its content (Moscovici, 1961). Social representations are used by individuals and groups to communicate, construct their identities, as well as guide and justify their practices. They are intimately connected with social practices, with action (Jodelet, 1991). Individuals and groups produce social representations through the active and contextualized reconstruction of socio‐historically established ideas, norms, and values (Moscovici, 1961). Therefore, social representations not only guide contextualized practices in the present but also express deep‐rooted symbolic structures in which they are anchored (Moscovici & Hewstone, 1983). Moreover, social representations provide the symbolic and figurative basis of relations of domination and counter‐domination, that is, they participate in the construction of ideology and counter‐ideology (Howarth, 2006).

Social representations theory posits that modern societies engender two main universes of social thought and practice, the reified and consensual universes (Moscovici, 2001). These universes are objective–subjective spaces that differ according to their source of validation, that is, expert/institutionalized (reified) or lay thinking (consensual; Moscovici, 2001). Scholars working with social representations theory have often investigated the ways in which knowledge from the “reified” universe of science comes to be located within the consensual universe of common sense, for example, in Moscovici's seminal study of the way in which novel and unfamiliar psychoanalytic ideas were transformed, made usable and familiar, for different groups in French society (Moscovici, 1961).

It is important to note here that social representations are sites of conflict and ambivalence as much as consensus (Rose et al., 1995) They are often characterized by cognitive polyphasia, defined as “the co‐existence of rarely compatible representations” (Wagner et al., 2000, p. 301). They are defined by argument, dilemmatic thinking, and resistance (Howarth, 2006; Moloney & Walker, 2002) and need to be understood as having particular political functions (Elcheroth et al., 2011; Howarth, 2006); common sense is ideological in that it can work to support or resist the status quo and invite social change (Castro & Batel, 2008).

In the context of citizenship, social representations structure the individual and group's ability, efficacy, and potential to engage in the everyday practice of claiming and enacting citizenship (Isin, 2009, 2012). For example, lay understandings of government and of the state enable and/or constrain particular forms of action and allow the performance of these actions by certain individuals and groups but not others. However, social representations are reified in institutions, government practices, and legal structures; they must be understood in ideological as well as socio‐cognitive terms. Further, from this perspective, we also acknowledge that understandings of citizenship are constantly in flux; its meanings are contested, resisted, and reshaped through action over time.

Weaving these influences together, we define citizenship here “as an everyday practice of invoking one's rights and making rights claims that position oneself and others as (legitimate) political subjects but which may also exclude others from political life” (Andreouli, 2019, p. 7). We foreground structural and ideological processes in our theoretical approach, informed by previous work examining the relationship between neoliberal capitalism and citizenship (Mitchell, 2016; Sparke, 2006), as well as by our understanding of social representation as a means of exerting and maintaining power (Howarth, 2006). Our attention to enactments of citizenship during the Covid‐19 crisis is important and necessary given the need to examine its forms during periods of political upheaval and transition, during which new forms of citizenship may be developed (Andreouli, 2019; Andreouli & Brice, 2021). Covid‐19 mutual aid groups presented a unique opportunity to investigate this possibility.

### UK Covid‐19 mutual aid groups

The pandemic motivated a significant community response across the United Kingdom and globally and one significant aspect of this has been the widespread proliferation of Covid‐19 mutual aid groups (Sitrin & Colectiva Sembrar, 2020). Some of these groups formed at the beginning of the pandemic, while others developed from pre‐existing organizations such as parish councils (Tiratelli & Kaye, 2020). Organized generally on a hyper‐local basis and via social media, they fulfilled practical tasks such as grocery shopping and collecting medication, but also provided invaluable emotional support and advice to members of the community, many of whom were struggling with physical and mental health issues as well as an economic disadvantage (Fernandes‐Jesus et al., 2021; Jones et al., 2020). Without mutual aid groups, vulnerable people would have had to rely on assistance from the local government, which was frequently perceived as too slow or inadequate in its provision (Tiratelli & Kaye, 2020). Research on participation in Covid‐19 mutual aid groups has suggested that their membership was disproportionately female (Jones et al., 2020; Wein, 2020), and that groups were more likely to be found in local authorities which were older, happier, and wealthier (Felici, 2020) or in places where there were higher levels of social capital and working‐age people had more time, chiefly via the government's “furlough” scheme (Tiratelli & Kaye, 2020). These findings suggest that, mirroring the country's broader experience of the pandemic, the community response was also shaped by pre‐existing economic and social inequalities.

Covid‐19 mutual aid can be considered to have a complex relationship with citizenship and political action, and particularly state‐centric accounts of both. This stems from the concept of mutual aid having its roots in the work of the anarchist theorist, Peter Kropotkin (1902, as cited in Kinna, 1995), and the various ways in which mutual aid groups have engaged with the term's ideological basis. From an anarchist perspective, mutual aid signifies a different model of society to the neoliberal capitalist status quo, one based on reciprocity and resistance to hierarchy and authority (Preston & Firth, 2020). Singh Dhillon (2020) noted that some UK mutual aid groups took a critical approach and understood mutual aid during Covid‐19 as an opportunity to radically restructure society over the long‐term, and what it meant to be a member of that society, in line with radical or anarchist principles. Members of these groups advocate a lack of engagement with and independence from the local authorities and police; their work is long‐term activism rather than short‐term crisis response. Mutual aid, from this perspective, is inherently political; it encompasses doing citizenship *without* and indeed *against* the state.

On the other hand, a recent survey found that members of UK Covid‐19 mutual aid groups are interested in politics and willing to engage in political action but view their mutual aid activities as apolitical (Wein, 2020). Other work has asserted that the membership of UK Covid‐19 mutual aid groups has been dominated by well‐meaning middle‐class Labour party (the main centre‐left opposition party) supporters who are ambivalent or hostile towards meaningful social change (Preston & Firth, 2020). Similarly, Singh Dhillon (2020) identified a further variant of UK Covid‐19 mutual aid, which functions as (likely short‐term) service provision/crisis response by partnering with third sector organizations, local government, and even perhaps the police. From this vantage point, mutual aid encompasses citizenship enacted *with* and *through* the state.

Most social psychological research on UK Covid‐19 mutual aid groups to date has applied a social identity approach. Work here has emphasized the importance of group dynamics for sustaining participation in mutual aid groups (Fernandes‐Jesus et al., 2021) and has demonstrated the positive impacts that these groups had on the well‐being of their members during the pandemic (Bowe et al., 2021; Wakefield et al., 2021), with some studies suggesting that these effects were moderated by perceived group politicization (*O'Dwyer, Beascoechea‐Segui, & Souza, í* 2021, Mao et al., 2020). Social identity theory has proven a useful theoretical lens with which to explore the phenomenon of Covid‐19 mutual aid, however, this approach has tended to focus on the *implications* of identification with and participation in mutual aid groups, without exploring the ways in which people understood and enacted mutual aid. This is needed given the phenomenon's complexity—its rootedness in anarchist political theory with its fundamental critique of statism, and the variability in terms of how this was enacted, calls for an examination of these *social representations* of mutual aid. Furthermore, as a political construct that would have been unfamiliar to those not versed in anarchist theory, it also offers a window into how knowledge associated with the reified universe was understood and put to work in the quotidian consensual universe (Moscovici, 1984).

### The current study

The current study applies a social representations approach to examine the following research questions: how was mutual aid understood and enacted by members of UK Covid‐19 mutual aid groups? How did they conceive of their groups’ characteristic values, processes, and relationships with other local structures or organizations? And what forms of citizenship were enacted by these groups? To explore these questions, we conducted semi‐structured interviews with 29 participants in these groups from across the United Kingdom.

## METHOD

### Participants

Twenty‐nine members of UK Covid‐19 mutual aid groups (15 female) participated in individual interviews between the 12th of May and the 3rd of June 2020. Demographic information about the participants is provided in Table 1. One participant was Irish and one Czech—all other participants were British. Participants were aged between 19 and 74 years (*M* = 44.69, *SD* = 15.79) and lived in England (16), Wales (8), and Scotland (5). We were unable to interview any participants of mutual aid groups in Northern Ireland due to significantly lower numbers of groups present in this region. All interviews were conducted either online or by phone and were audio‐recorded. Each of the authors was involved in interviewing, with the majority (16) being conducted by the second author.

**TABLE 1:** Demographic characteristics of participants

Pseudonym	Gender	Age	Nationality	UK region
Alasdair	Male	65	British	South West England
Alice	Female	45	British	Wales
Andrea	Female	51	British	East Midlands
Bethan	Female	40	British	Wales
Charlotte	Female	28	Czech	Wales
Chris	Male	26	British	Greater London
Conor	Male	26	British	South East England
Daniel	Male	54	British	Wales
David	Male	59	British	Wales
Deborah	Female	41	British	Wales
Diarmuid	Male	54	Irish	Scotland
Elaine	Female	53	British	South West England
Gavin	Male	54	British	North West England
Grace	Female	25	British	Greater London
Hannah	Female	26	British	North West England
Jack	Male	21	British	Greater London
Katie	Female	27	British	Greater London
Malcolm	Male	43	British	Greater London
Mandy	Female	49	British	Scotland
Marie	Female	57	British	Scotland
Michael	Male	70	British	Greater London
Nick	Male	43	British	East Midlands
Peter	Male	40	British	Wales
Poppy	Female	19	British	Wales
Robert	Male	66	British	Scotland
Sam	Male	33	British	West Midlands
Steven	Male	74	British	Scotland
Stuart	Male	46	British	South East England
Yvonne	Female	41	British	Greater London

All participants were recruited through their recent completion of an online survey, at the end of which participants were invited to provide their email address if they were interested in a follow‐up interview to provide further detail. This survey also examined experiences of participation in UK Covid‐19 mutual aid groups: it comprised questions that measured their experiences of and perceptions of their mutual aid group, their views on various social and political issues, their perceptions of Covid‐19, well‐being, and demographic characteristics. We recruited participants to this initial survey through invitations distributed by the first author on social media, chiefly Facebook/WhatsApp groups, depending on the primary method of contact for the group advertised on the central organizing website (Covid‐19 Mutual Aid, 2020). From this pool of potential interviewees, we invited participants to an interview in a way that maximized the diversity of the sample in terms of gender, age, and location (see Table 1). All participants were assigned pseudonyms for the analysis.

### Interview context

Interviews were conducted during the first nationwide “lockdown,” which was announced by UK Prime Minister Boris Johnson on the 23rd of March. On the 10th of May, Johnson announced some very modest easing of restrictions: the most notable of these was encouraging those unable to work from home to return to work. Therefore, all participants were interviewed in the immediate aftermath of this modest easing of restrictions.

It may also be relevant here to provide further relevant contextual information in relation to the UK government's Covid‐19 response. At the end of March 2020, the UK government allocated emergency funding of £3.2 billion to local authorities, as well as £5 billion in cashflow support (Ministry of Housing, Communities & Local Government, 2020). Local authorities supported their communities with the provision of hardship grants for individuals (generally with strict eligibility criteria), as well as small grants for voluntary organizations, such as mutual aid groups. However, this should be contextualized by recognizing the significant financial pressures that local authorities have experienced due to budget cuts by the UK government over the past decade of austerity programmes, a drop in funding estimated at being nearly £16 billion by 2020 (Local Government Association, 2018).

### Procedure

Prior to the interview, participants read information about the study and provided consent and demographic information via an online survey hosted on Qualtrics. Most interviews (21) took place online via the web‐conferencing platform, Zoom, however other participants elected to use other mediums and platforms—phone call (4), Skype (2), Microsoft Teams (1), and Google Meet (1). Interviews lasted between 19 and 82 min (*M* = 50.00, *SD* = 15.02) and were recorded using the in‐built recording function of the web‐conferencing platform or using a call recorder application on the researcher's mobile phone. The semi‐structured interview schedule addressed the following broad topics: (1) their perceptions of the mutual aid group (i.e., its origin, structure, processes, and activities); (2) the way in which the mutual aid group interacted with other actors in the crisis (i.e., the local council, food banks, etc.); and (3) their experiences of participation in the group and any challenges faced. The study received a favourable ethical opinion from the ethical review board of Kingston University, London.

### Analysis

The audio files were transcribed verbatim and analysed using reflexive thematic analysis (Braun & Clarke, 2006, 2019). In contrast to other types of thematic analysis underpinned by post‐positivist assumptions, in reflexive thematic analysis, themes are defined as “creative and interpretive stories about the data, produced at the intersection of the researcher's theoretical assumptions, their analytic resources and skill, and the data themselves” (Braun & Clarke, 2019, p. 594). We adopted a primarily inductive, theoretically sensitive approach to the analysis, which was underpinned by a critical realist epistemological position (Bhaskar, 2013; Fletcher, 2017). Themes were developed based on the participants’ accounts (the main categories were not pre‐determined) and our theoretical framework (attending to contents and implications relevant to the social representation of mutual aid). The analytic process is comprised of four stages:

1. Based on initial notes and a group discussion of the interviews, we developed a coding frame to enable us to address the research questions. Following our research questions, we coded all data which referred to (1) *perceptions of the mutual aid group*—its structure, processes, and ingroup values and norms; (2*) outgroup perceptions and relations*—the way in which other groups respond to the crisis (e.g., local councils and charities) were described and how relationships with these groups were characterized.
2. We divided the 29 transcripts between us and individually coded our assigned transcripts using the qualitative analysis software NVivo, using the previously developed coding frame.
3. When coding was complete, we discussed and reviewed the codes which we had collectively generated. The three separate NVivo files we had used were merged into one. Codes were grouped, merged, and discarded after discussion. In line with our chosen method and epistemological position, our objective here was not to seek agreement or consensus across analysts (e.g., via assessments of intercoder reliability), but rather to develop a rich and nuanced analysis, which stuck closely to the data.
4. The first author then developed the thematic structure from the list of codes. Codes were grouped by shared meaning to form superordinate themes, with superordinate themes then joined to create more interpretative and abstract “stories” about the data.
5. We met again to discuss the themes developed by the first author and made final amendments to the structure by reviewing it on NVivo. The final thematic structure is displayed in Table 2.
6. The first author then wrote up the findings in a detailed analysis.

**TABLE 2:** Thematic structure developed through the thematic analysis

Codes (Level 1)	Subthemes (Level 2)	Themes (Level 3)
Independence from formal authorities Ineffective response of the council Efficiency Competition Collaboration Motivations Distrust Misunderstandings	a. Relationships with external groups	Mutual aid within/against
Solidarity Universality Kindness	b. Other‐focused values	
Crisis response is not a political movement Mutual aid is inherently political Some forms of mutual aid are more political	a. Politicisation of mutual aid	Avoiding politics
Inclusivity Follow internal codes	b. Ingroup norms and values	
Skills‐based Multiple roles Levels of organization	a. Complexity	Organized units
Hierarchical Flat structure Growth process Flexible	b. Horizontalism	

### Findings

Before describing the findings from the thematic analysis, we provide some details here on the wide range of actions that our participants linked to their groups. These actions included establishing and maintaining communication channels with the public, through support hotlines, flyers, social media, and in one case, a radio station. Participants reported engaging in a range of actions related to the provision of food (e.g., grocery shopping and delivery and preparation of food parcels). Collecting and delivering medicine was also commonly reported as well as providing social and emotional support (e.g., befriending services). Less commonly cited actions included dog walking, loaning items, posting letters, and providing financial support to ensure people's access to electricity and the internet. Overall, the actions ensured that the public (especially vulnerable people) could stay at home and protect themselves from the coronavirus. Some actions had a direct health focus, for example, promotion of social distancing in shops, and the provision of personal protective equipment. Finally, another broad array of actions related to the management of the mutual aid group, for example, creating and managing social media accounts, holding meetings, fundraising, and encouraging volunteers.

The thematic structure developed in the analysis is displayed in Table 2. We developed three themes through the analysis—*organized units*, *mutual aid within*/*against*, and *avoiding politics*. These themes reflect participants’ accounts of their mutual aid groups in terms of their structures and processes, characteristic values, and their groups’ interactions and relationships with external organizations. Each of these themes will now be described and interpreted in turn.

### Organized units

This theme relates to the efficient and complex processes and non‐hierarchical structure of mutual aid groups, which featured, in most participants’ accounts as a direct comparison to the ineffectiveness of the local government response to Covid‐19. Participants described their mutual aid groups as nimble, organized, and effective units. The efficiency of the group was facilitated by a panoply of web‐based tools (e.g., WhatsApp, Google Forms, Zoom, etc.):

Yes, the chair of the organising committee sends out a poll, an online poll and you check when you’re available. And then they work out the rota then send an email back telling you or showing you the rota and when you’ve been chosen to help. (Alasdair, 65, South West England)

Efficiency seemed to be the ultimate priority for many groups: “our first priority of what we're doing is to get people help in the fastest and most efficient way possible” (Sam, 21, Greater London). The group's efficiency and nimbleness were frequently contrasted with the inefficiency and bureaucracy of the local government response: “if we'd left it to the local council, they'd still be working out what colour to print the leaflet at the moment” (David, 59, Wales). This served to underscore the collective efficacy of mutual aid groups.

Furthermore, mutual aid groups were frequently described as very complex systems—volunteers were matched to tasks depending on skills, interests, or availability and activities were for the most part organized on a geographical basis (e.g., voting wards and streets):

We have officer teams that are split up between, let me remember, general officers. So, as I said, that’s people who coordinate between the different groups. Task officers who collate and then send out tasks to be done by volunteers. Ground officers whose job is to organise our structure and to get new volunteers into the system and to do introduction meetings and all that. Outreach officers, that would effectively be the marketing wing of the group and their job is to coordinate with the council and other groups, and also to expand our reach into the community. I’m forgetting one. There’s GDPR, data and finance, and the legal team. But that’s just basically compliance. (Sam, 21, Greater London)

Notable here and frequently across the transcripts is the way in which the structure of the mutual aid group is anchored in the representation of a corporate entity. Jack's invocation of “GDPR, data and finance, and the legal team” and “outreach” is clearly located within the register of the modern managerial/corporate environment. Elsewhere, Yvonne (41, Greater London) explicitly described her mutual aid group as analogous to “a normal company.” This functions to locate mutual aid within a sphere that would have been familiar to the professionals who participated in these groups, but it may also constrain possibilities for action outside of neoliberal parameters.

Participants also frequently discussed the horizontal/hierarchical structure of their groups. Aligned with an anarchist conception of mutual aid, many participants characterized their groups as non‐hierarchical: “We're non‐hierarchical as well so we don't have a leader, we have several officer groups that all try and interlink” (Jack, 21, Greater London). Participants described their group's organizational structure as “loose” (Michael, 70, Greater London) and “fluid” (Malcolm, 43, Greater London). Leaders, such as they were, were generally referred to with terms such as “facilitators” (Katie, 27, Greater London), “coordinators” (David, 59, Wales) or “admins” (Hannah, 26, North West England), which were perceived as necessary mostly because of the range or scale of activities in which groups were participating: “just because a borough is a really big area to organise” (Katie, 27, Greater London). Leadership in mutual aid groups was characterized as happening organically, as something which was dependent on the experience and availability of group members, rather than a desire for control or authority.

### Mutual aid within/against

Within participants’ accounts, there was divergence in terms of the way in which their practice of mutual aid was described as existing within or independently of formal authorities and structures. Some participants reported that their groups worked closely with local government, with councillors frequently playing an active role in the group's activities. The relationship with the council was frequently described in instrumental terms: displaying the local council's logo on the group's advertising materials “gave it a validity” (Mandy, 49, Scotland) and using council resources could be otherwise useful: “the parish council has a secure server so it, people's details are on it, so we did it through that” (Stuart, 46, South East England). In other cases, collaboration with the local council directly facilitated the group's access to those most in need: “There was a need for there to be a more umbrella organisation for it. Because however much people wanted to help, they couldn't necessarily contact the people that needed the help the most” (Bethan, 40, Wales).

However, other participants reported negative relationships with their local councils or were ambivalent about collaboration. The following excerpts illustrate the way in which participants described their relationship to the council and drew upon in‐group values to emphasize both the distinctiveness and efficacy of their mutual aid groups:

So, the council’s attitude is that people need to be referred to food banks by one agency or another or the council. So, you have to prove that you’re in need of food and so on. Whereas our attitude and the community kitchen’s attitude is if you say you need food then you get food. And it’s no questions asked either about sort of whether you really need it or about things like migration status which can be an issue as well. (Michael, 70, Greater London)

***

Andrea So, now I’ve got a glut of food in my hallway and we’re thinking of setting up a little food bank or something in the community centre here where I am, but I don’t know how that’s going to work. I’ve been in touch with X Council about it, about grants to help us set that up, and they said yes, okay, so as long as you don’t take it to the next town along, they’re not allowed to use it. It’s like, all right, okay, bureaucracy again. And how would you stop that anyway? It’s only three miles, the next town, how would you stop somebody from three miles away not having anything if they were starving, you wouldn’t, would you?

I No, of course not.

Andrea You’d go, yes, yes, Councillor, and then you’d give them it anyway, wouldn’t you, you wouldn’t see anybody hungry.

(Andrea, 51, East Midlands)

Michael contrasts the conditional nature of support linked to the council (“you need to be referred to food banks”) with that of the mutual aid group (“no questions asked”). Through this contrast, he legitimizes the activities of his group and underscores its distinctiveness. Similarly, Andrea compares the geographical condition of support required by the council and the “bureaucracy” of this approach, with the position that “you wouldn't see anybody hungry.” Salient in these excerpts and across the data are other‐focused values of solidarity, kindness, and trust. These values, by emphasizing the fundamental humanity of all people and their entitlement to unconditional support, buttress a conceptualization of citizenship that is not tied to factors such as legal status or a formal assessment of need. It rejects a definition of citizenship as something which must be earned (Andreouli & Dashtipour, 2014) either via legal status or engagement with state structures or processes (i.e., the highly conditional social welfare system)—everyone is entitled to help. It is similarly oppositional to neoliberal citizenship which determines an individual's worth based on their economic contribution, that is, as consumers or workers.

A similar tension was evident in the way in which participants described their group's relationship with the local police. While a few participants actively collaborated with police officers (e.g., inviting them to organizational meetings as community representatives), other accounts reflected ambivalence about such collaboration:

Grace And, also, they were talking about the police and stuff as well. Like, I didn’t understand that at all. But I also felt like I didn’t want to ask because it was like a group of seven of us in this caseworker meeting and the person running the meeting was like “we don’t work with the police”. And, I would have wanted to ask why, but I assumed something like political, so I didn’t. I didn’t really want to ask in that setting. I’m sure I could email. Well, I might actually email and be like why? Because if I was doing casework with someone and they wanted to ask. Apparently, I can’t be involved with the police and I don’t really know why. They just said it and it was very clear. They were just like “we don’t work with the police”.

I Interesting.

Grace Yes. Because they’ve got a thing on their website now as well. I’ll just go on it. I felt slightly uncomfortable after the caseworker meeting because it just did come out a bit more maybe, yes, how political it could be to be a part of a group like this and that’s not really the reason I joined it.

(25, Greater London)

Recounting her attendance at a training session to support her role in the group, Grace reports being baffled by the position taken by her group on engagement with the police, sensing that it was “political” but being unable to query it (“I didn't really want to ask in that setting”). Elsewhere in her account, Grace stated that she joined the group “to help with the Covid stuff, like to organize support like that.” Evident here is the interface between two oppositional representations of mutual aid, which is salient through ideas about normative social practices (“we don't work with the police”). Mutual aid from a radical/anarchist perspective necessitates complete independence from state authorities, including the police. There is a tension in Grace's account that stems from the conflict between her conceptualization of mutual aid (grounded in a traditional charity model), and those of the organizers of her group, who conceptualize Covid‐19 mutual aid as a means to radically restructure society *as well as* help people survive self‐isolation. This excerpt appears to reflect disagreement about the purpose and form of mutual aid across and within UK Covid‐19 mutual aid groups (Preston & Firth, 2020; Singh Dhillon, 2020), and more broadly to tension between doing citizenship within or against state‐sanctioned contexts (Miraftab, 2004).

### Avoiding politics

This theme referred to the tension between practicing a political and critical form of mutual aid, on the one hand, and the need to be inclusive, on the other. One of the core ingroup values linked to mutual aid groups across participant accounts was inclusivity. This was evident in frequent discussions of the potential problems associated with the use of social media (“they might be elderly, and they might not be familiar with technology, and technology has been a massive tool for communication during this pandemic,” Conor, 26, South East England) and the need to ensure the membership of mutual aid groups reflected the areas in which they were located (“X Mutual Aid has been trying to increase diversity of the actual volunteers as well,” Grace, 25, Greater London), for example.

However, the over‐arching value placed on inclusivity, the need for “maximum community engagement” (Malcolm, 43, Greater London), meant that most participants described their mutual aid groups as spaces in which politics would generally be avoided. This had benefits in terms of reaching people of different ideological positions, however, it also presented difficulties for the participants themselves. The following excerpts illustrate this:

I think the advantage is that we bring in people who would otherwise be turned off. So we can really bring people in who have a more conservative mindset etc. Those who wouldn’t want to be part of a group that pushes a particular political agenda, but who are still very interested in helping neighbours and trying to get together through the crisis. So we feel our approach is, in that sense, more inclusive. (Malcolm, 43, Greater London)

***

You know, we all have our opinions on how things are going on, and particularly with all the stuff with Dominic Cummings and things coming out, and you know, it’s been very difficult to bite your tongue on some of these things. But we’ve tried to keep the group non‐political so that the aims of what it’s doing aren’t then being diluted by arguments and negativity, and it kind of just remains positive. (Sam, 33, West Midlands)

The strategic benefit of avoiding politics in mutual aid is clear in both statements—it makes mutual aid attractive to people with diverse views, who see their activities as “helping neighbours,” and it keeps mutual aid “positive.” However, as evident in Sam's account, this could be difficult for participants, who were in the main politically active and engaged individuals, and often had strong and critical opinions about the UK government's handling of the pandemic (“difficult to bite your tongue”).

While participants described a strategically apolitical type of mutual aid, nevertheless many also recognized their actions as *inherently political*, as the following excerpt illustrates:

We’ve just decided that we're going to help the people who need help, and if someone chooses to take that as a declaration of political loyalty, that's their issue, because we're not. But we are making, I would say, a political choice that everyone is worthy of help. Everyone is worthy of feeling like a human being, and nobody can tell us that actually, no, you can't help this person, or this person shouldn't be worthy of help. Because if they do, we'll just ignore them and do it anyway. Try and stop us. (Katie, 27, Greater London)

Mutual aid here is described as outside party politics, but it is far from apolitical. It is grounded in a fundamentally humanist perspective tied to the value of respect for human dignity (“everyone is worthy of feeling like a human being”). It pays no attention to considerations of “deservingness” (“we're going to help the people who need help”) and it does not need to be endorsed by the state (“we'll just ignore them and do it anyway”). Evident across all participants’ accounts was a similar process of claiming space to carry out mutual aid, irrespective of whether it was endorsed by existing local structures. Thus, whether these groups were explicitly presented as political or not makes no difference: they had an inherent critical and political remit because of the way in which they functioned to highlight the fundamental anti‐humanity of the state, exemplified by its response to the pandemic.

## DISCUSSION

Our analysis provides insight into the internal dynamics and organization of UK Covid‐19 mutual aid groups as well as their typical ingroup values. Two central tensions appeared to characterize mutual aid groups, particularly that between collaboration with existing organizations and structures, and resistance to this, and between maximizing group inclusivity and sustaining political critique.

Both of these tensions, we argue, invite us to consider the relationships between Covid‐19 mutual aid, political critique, and the status quo. Some groups worked within existing structures in the community (e.g., parish councils and resilience groups) or collaborated with local authorities such as the council or police. For other groups, working outside or against local authorities was either a decision that was consciously taken due to perceived ideological incompatibilities with mutual aid, or was imposed on groups by the local authorities’ failure to interact with them. Thus, mutual aid was variously performed in either the “invited” or “invented” spaces of citizenship (Miraftab, 2004). Participants employed the social creativity characteristic of consensual universes (Moscovici, 2001) to propose principles and practices of citizenship independent of institutionalized authority. Further, it speaks to an identified tension in how citizenship is conceptualized, in terms of either a habitual practice that is afforded and facilitated by state institutions, or as a transgressive practice that works to trouble pre‐existing ways of doing citizenship (Isin, 2009). Our finding here highlights the continued need to move away from state‐centric accounts of citizenship, to examine the ways in which people construct, enact, and claim citizenship for themselves.

Relatedly, we turn to the tension which was evident in the data between maximizing inclusivity and sustaining political critique. All study participants were on the left of the political spectrum; many were members of political parties and trade unions. A survey of participants in UK Covid‐19 mutual aid groups (O'Dwyer, 2020) reported that these groups were over‐whelming left‐wing in political orientation. It was surprising then throughout the data the extent to which participants described their groups as apolitical spaces. The political nature of mutual aid appeared to stem from their prefiguration of an alternative model of society based on values such as kindness and solidarity, rather than its explicit critique of existing institutions and practices, such as the highly conditional social welfare system. However, even with this broader prefigurative quality, the strategy employed by organizers—to keep politics out of mutual aid—is notable. It can be seen to function through a neoliberal logic that has worked to recast people as consumers instead of citizens (Clarke et al., 2007)—atomized individuals at the service of capital, divorced from the relationality of the public sphere. This finding supports an interpretation of the social representation of mutual aid as polyphasic (Wagner et al., 2000), in which contradictory and oppositional elements co‐exist.

The degree of organizational complexity of mutual aid groups, and the breadth of activities in which they were involved, was striking. Mutual aid groups were characterized as complex and sophisticated operations which made pragmatic use of their members’ skillsets and organizational capacities. Leadership was an organic response to the *scale* of the challenges which mutual aid groups were presented with, which was combined with an active commitment to an absence of hierarchy. Participants’ talk about their groups in many instances was anchored in representations of the corporate structure, referencing terms such as GDPR and “outreach.” Both the centrality of efficiency to representations of mutual aid and the way in which these representations were anchored in representations of the corporate structure show how mutual aid was understood and enacted through a neoliberal lens. However, while this representation was made familiar with neoliberal values and practices, it also presents the possibility of resistance, a troubling of the status quo. We interpret this use of neoliberal ideas and practices, and their accommodation within the social representation of mutual aid, as pragmatic and strategic (Changfoot, 2007): it co‐opts this discourse to resist it, to gain legitimacy for a group that prioritizes solidaristic rather than individualistic ways of structuring society. In effect, we argue that this provided participants with the register to claim the efficacy and efficiency of their groups while they simultaneously resisted the type of citizenship it signified.

During the pandemic, and in direct response to the mobilization of mutual aid groups across the country, the Conservative MP Danny Kruger (and former aide to former Prime Minister David Cameron) launched the New Social Covenant Unit, which made the case for an expansion of localism and “community power” as a way of reducing economic inequalities and facilitating community cohesion. He called for a new social covenant between individual citizens, civil society, and the state—“a mutual commitment by…each to fulfil their discrete responsibilities and to work together for the common good of all” (Kruger, 2020, p. 12). This is intelligible with recent work on citizenship in the United Kingdom, which suggests that the national government emphasized other‐focused values and actions at the early stage of the pandemic (Andreouli & Brice, 2021). Our analysis suggests that there is a clear appetite and capacity to bolster “community power” however it cautions against “bureaucratizing” such endeavours and advocates minimal, supportive facilitation by the local and national government. Simultaneously, however, we question to what extent the Conservative government may actually appreciate and so facilitate increased active citizenship and “community power” when our analysis suggests that, in some cases, this power may be turned towards government critique, particularly in relation to bureaucratic, centralized, and hierarchical structures and processes.

Relatedly, we posit that one of the drivers of reported tensions between local councils and mutual aid groups could plausibly be traced to the different models of citizenship inherent in their activities, with the former employing a conceptualization dependent on a determination of legal status, “deservingness” (Andreouli & Dashtipour, 2014), or on a geographical basis, and the latter a broader conceptualization which offers unconditional support from a starting position of trust. From this perspective, the social practices associated with mutual aid are *fundamentally* a critique of the status quo as they demonstrate the possibility of an alternative, more compassionate, social welfare system.

The scale of the activities in which participants reported being engaged in some instances resembled a parallel social welfare system, including financial assistance, emotional support, and even a public health component in some cases. The analysis clearly showed that mutual aid groups extended their support to those unable to receive assistance from the local or national government due to eligibility criteria. Our analysis thus supports the assessment of Tiratelli and Kaye (2020) that these groups were indispensable in terms of people's ability to manage the first UK “lockdown.” Our analysis goes somewhat further perhaps, however, and suggests that, were UK residents to have relied solely on local government for support, they would for the most part have been totally unable to remain in their homes. Relatedly, we need also to acknowledge (and investigate) the political, social, and economic context in which mutual aid was deemed necessary, which motivated our participants to perceive a vacuum of support and a need for local collective action to address it.

There are a number of limitations to the current study. First, the participants, as self‐selecting, were likely to have had more clearly defined views on mutual aid and were, for the most part, involved in the administration and organization of their groups. The majority of participants were also those who had been involved in supporting other individuals and had not themselves received mutual aid. Given that previous qualitative studies on Covid‐19 mutual aid have also drawn upon interviews with organizers (Fernandes‐Jesus et al., 2021; Mao et al., 2020), future work on mutual aid groups should include participants at different levels of involvement in the group as well as beneficiaries of aid. Second, none of our participants subscribed to an anarchist interpretation of mutual aid, though all were aware of the term's provenance. Clearly, many *did* subscribe to this particular interpretation, and indeed may have felt that Covid‐19 mutual aid was co‐opted by middle‐class concerns and values (Preston & Firth, 2020). Anarchist participants in mutual aid groups were approached to participate but none did. Our experiences during data collection suggest that this could be due to this group's ideologically motivated opposition to participation in academic research. This needs to be kept in mind when we are tempted to generalize about UK Covid‐19 mutual aid in the absence of anarchist and radical voices. Third, mutual aid has been a commonplace response to Covid‐19 across the world (Sitrin & Colectiva Sembrar, 2020); future research would do well to examine its forms in different national contexts.

In summary, this study characterizes UK Covid‐19 mutual aid groups as organizationally complex, efficient, and associated with organic and porous leadership. As a way of doing citizenship, Covid‐19 mutual aid provided a means for people to participate in their local communities, without the bureaucratic constraints usually imposed by charities and voluntary organizations. Operating on the principles of solidarity, kindness, and trust, their activities offered a glimpse of an alternative way of structuring society with different ideas about what citizenship might mean in such a society. Their prefigurative activities during Covid‐19 should, we argue, be considered as political critique, as well as effective altruism. The impact of these new experiences of a *different type* of citizenship, acquired through Covid‐19 mutual aid, may have significant effects on future modes of political participation and engagement in the United Kingdom.

## CONFLICT OF INTEREST

The authors have no conflicts of interest to declare.

## AUTHOR CONTRIBUTION

**Emma O’Dwyer:** Conceptualization; Data curation; Formal analysis; Funding acquisition; Investigation; Methodology; Project administration; Supervision; Writing – original draft; Writing – review & editing. **Luiz Gustavo Silva Souza:** Conceptualization; Data curation; Formal analysis; Methodology; Project administration; Writing – review & editing. **Neus Beascoechea‐Seguí:** Formal analysis; Investigation; Methodology; Project administration; Writing – review & editing.

## Data Availability

The data that support the findings of this study are available from the corresponding author upon reasonable request.
